# Case Report: Cognitive Work Hardening for Return-to-Work Following Depression

**DOI:** 10.3389/fpsyt.2021.608496

**Published:** 2021-04-12

**Authors:** Adeena Wisenthal

**Affiliations:** ERGO-Wise, Ottawa, ON, Canada

**Keywords:** cognitive work hardening, depression, return-to-work, mental health, disability, rehabilitation, occupational therapy

## Abstract

The growing number of mental health disability claims and related work absences are associated with a magnitude of human, economic and social costs with profound impact on the workplace. In particular, absences due to depression are prevalent and escalating. There is a need for treatment interventions that address the unique challenges of people returning to work following an episode of depression. Occupational functioning often lags depression symptom improvement which necessitates targeted treatment. Cognitive work hardening (CWH) is a multi-element, work-oriented intervention with empirical research supporting its role in return-to-work following a depressive episode. This case report details the use of CWH to prepare an individual to return to work following a disability leave due to depression. It illustrates how CWH bridges the functional gap between being home on disability and returning to competitive employment. The client presented is a 50 year old divorced woman who had been off work for approximately 2 years for depression precipitated by the terminal illness of her mother. She participated in a 4 week CWH program which addressed fatigue and decreased stamina, reduced cognitive abilities, outdated computer skills, and heightened anxiety. Work simulations enabled the rebuilding of cognitive abilities with concomitant work stamina; task mastery bolstered self-confidence and feelings of self-efficacy; and coping skill development addressed the need for stress management and assertive communication strategies. By program completion, the client's self-reported work ability had increased and both fatigue and depression symptom severity had decreased. Clinical markers of work performance indicated that the client was ready to return to her pre-disability job. Three months after completion of CWH, the client reported that she was at work, doing well and working full days with good stamina and concentration. This report provides insight into how CWH can be applied to return-to-work preparation following depression with positive outcomes.

## Introduction

Depression is a leading cause of disability worldwide and one of the most common mental disorders in the workforce (1, 2). With its negative impact on work performance, including impaired cognitive functioning and reduced stamina, disability rates are increasing with protracted sick leave and difficult return-to-work (RTW) trajectories (3–5). While off work on disability, a person typically receives treatment (e.g., psychotherapy, medications) to alleviate depressive symptoms (6, 7). However, clinical improvement does not necessarily result in full recovery of job performance, often due to residual effects of the depression and associated functional impairments such as reduced concentration, decision-making, and task performance (8–11). There is a need to rebuild work capacity by targeting occupational function, identifying and addressing RTW barriers, and planning for a supportive and graduated work re-integration (6, 10, 12). The need for work-focused interventions has also been linked to the functional requirements of work being significantly more demanding than those in the home or community (13) and the reality that some employers are reluctant to have an employee return to work who is not functioning at a minimal requisite level (14).

**Figure 1:**
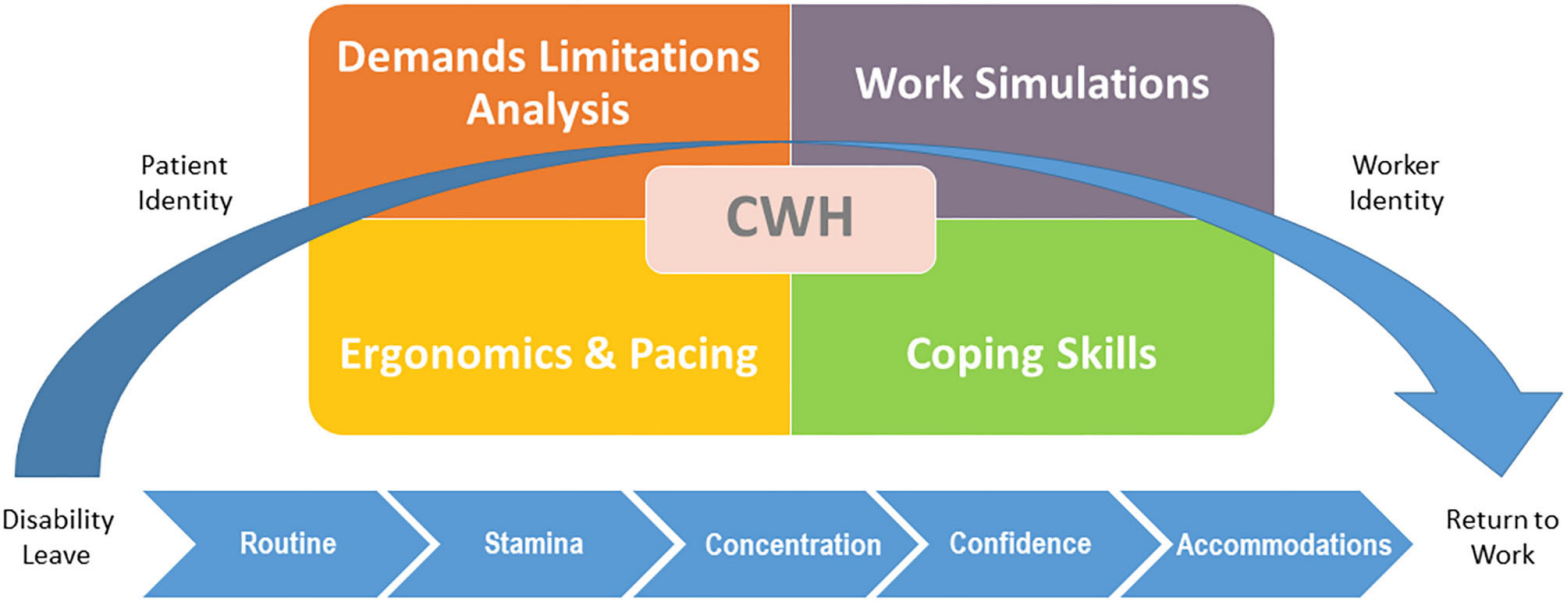
Elements of cognitive work hardening. Wisenthal (15), © Adeena Wisenthal, 2020. All rights reserved.

Cognitive work hardening (CWH) is a multi-element, occupationally-based intervention (see Figure 1) that bridges the gap between depression symptom improvement and work functioning (16). It is derived from the more classical work hardening, an evidence-based treatment intervention that has largely been provided by occupational therapists to restore work capacities mostly for people with physical injuries and pain (17–19). CWH is tailored for people recovering from depression to restore work capacity through the use of work as a treatment modality. Graded work tasks are used to progressively rebuild the mental energy and cognitive abilities needed for RTW. Task mastery promotes self-confidence and feelings of self-efficacy. Education on coping skills helps build resilience by equipping clients with effective communication strategies to handle interpersonal relationships and conflict. This approach aligns with research findings that conflict at the workplace is predictive of recurrent sickness absence among workers returning following a mental health disorder such as depression (20). CWH has been scientifically studied (21, 22) with research findings supporting its role in RTW following depression.

The purpose of this case report is to build on this quantitative and qualitative research by offering a concrete example of how CWH can be utilized in clinical practice. Specifically, the practitioner is provided with a detailed case illustration of a typical CWH client, a guided application of the multiple elements of the intervention, and a RTW outcome typical of the intervention. Moreover, the client feedback (based on experience in the intervention) adds important context. In so doing, this case report complements evidence-based research and offers a window into a novel intervention with the intent of advancing knowledge—key strengths of the case report format (23, 24). This report responds to the call for enhancing research utilization capacity through knowledge transfer (25, 26) by providing an in depth look into a client that participated in a CWH intervention with positive RTW outcomes. Insight into the processes of the intervention, their utilization in practice, and the meaning of the intervention to a particular client inform occupational therapy practice and other stakeholders in the RTW domain.

## Case Description

### Patient Information

This case presents a 50 year old divorced woman who was off work due to depression in response to her mother's cancer diagnosis and ultimate passing. The client had been off work for approximately 2 years prior to her referral for CWH. She had been off work once before (for 6 months) 1 year prior to the current leave, again due to her emotional struggles related to her mother's diagnosis. The client was being followed by her family physician and her psychiatrist, each on a monthly basis. She had also seen a psychologist bi-weekly for 18 months. The client was prescribed Bupropion and vitamins.

The client was referred for CWH at ERGO-Wise, an occupational therapy private practice in Ottawa, Canada that specializes in workplace mental health and RTW preparation. The client had graduated from a University level nursing program and had worked in the healthcare field for over 30 years, including 20+ years in her pre-disability adjudicator job at a government agency. She had been divorced for 20 years after having been in an abusive marriage. She noted difficulty with authority and conflict as a result of this relationship. The client lived on her own in close proximity to her one daughter. Regarding engagement in meaningful activity, the client reported reading, knitting, and going for daily walks.

**Figure 2:**
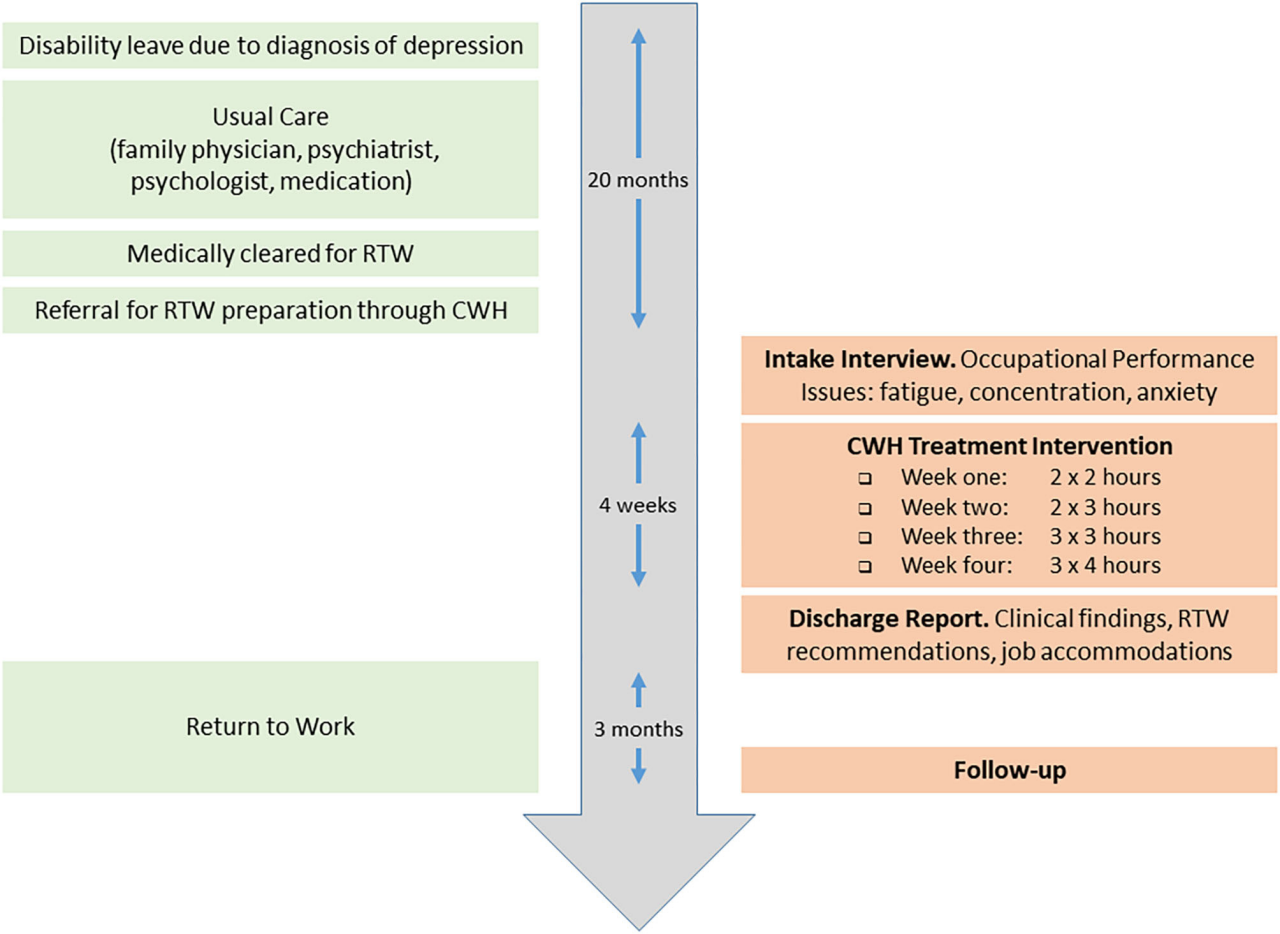
Client care timeline.

Consistent with CWH protocol, the client had received medical clearance to return to work (at the time of the referral) due to a reduction in her depression symptoms and an improvement in her mood state. Regarding RTW barriers, the client reported: (i) decreased cognitive abilities (e.g., reduced concentration); (ii) fatigue and decreased stamina; and (iii) the need to update her computer skills. The client also reported that she lacked assertiveness and felt threatened by authority and/or conflict situations. This manifested in heightened anxiety with interpersonal relationships for fear of potential discord. Figure 2 summarizes the intervention timeline.

### Treatment Intervention

Following referral to CWH and with the client's signed consent to proceed with an intake interview and to release information to the referring person, the treating occupational therapist conducted a semi-structured interview to obtain background information about the client's work history, disability trajectory, and occupational performance issues. The interview helped the therapist understand the client's occupational performance challenges and RTW obstacles while it gave the client the opportunity to better understand the CWH intervention and the therapist's role in the RTW process. Through this intake process, the therapist made a positive determination about the client's suitability for CWH.

The CWH intervention was provided in a simulated work environment located in an office building conveniently situated for car or public transportation access. The office suite resembled a typical work environment with several workstations each equipped with a computer, keyboard/mouse, height adjustable monitor, and fully adjustable chair. The intervention was 4 weeks in duration with the following structure: Week One: 2 ×2 h; Week Two: 2 ×3 h; Week Three: 3 ×3 h; Week Four: 3 ×4 h; totaling 31 h.

The CWH intervention was provided by a registered occupational therapist aptly qualified for this role given that the intervention's underlying principles are cornerstones of the occupational therapy profession. These include assessment of occupational functioning, work demands analysis, and functional gap mitigation (27, 28). Furthermore, the intervention's use of work as a treatment modality is a well-grounded occupational therapy strategy (29). The occupational therapist's skills in clinical observation and clinical reasoning (30), together with expertise in mental health (31) and knowledge of fatigue/pain management strategies (e.g., pacing, ergonomics) (32) well-position the occupational therapist for this key role in RTW preparation.

On the first day of CWH, the client completed three self-report questionnaires that address constructs that can relate to RTW outcomes (21). The questionnaires were the Work Ability Index (WAI) (33), the Multi-Dimensional Assessment of Fatigue (MAF) (34), and the Beck Depression Inventory II (BDI-II)^1^ (35). These three questionnaires were also administered on the last day of the CWH program serving as pre- and post- self-report measures of change following participation in CWH.

The WAI is utilized for the self-assessment of work ability and the evaluation of the effects of intervention programs on work ability (33). The MAF measures four dimensions of fatigue (e.g., severity, distress, timing, degree of interference in activities of daily living) (34). The BDI-II is a widely used screening measure of depressive symptoms (35).

The client's first work task was to complete a job description which re-connected her to her pre-disability work. This task required concentration and memory and although these cognitive abilities had atrophied while being on disability, the client noted that it felt good to use them again, even if not at full capacity. The job description helped the occupational therapist gain a better understanding of the client's job demands and work tasks. Through job analysis, the therapist determined the cognitive abilities the client required to meet her job demands setting the stage for designing work simulations that modeled the client's job tasks and/or the cognitive abilities needed to perform her job. Work simulations are the cornerstone of the CWH intervention. The occupational therapist ensured they were relevant and meaningful to the client and to her work. Such customization of work tasks renders the work more “real,” even if simulated.

In order to address the client's concern regarding outdated computer skills, the occupational therapist incorporated work tasks that required the use of Microsoft Office (e.g., Word, Excel) as well as Internet searches to assist the client in becoming more current in technology and various applications she would need once back at work. Meaningful and relevant work simulations included having the client research new policies/procedures related to her workplace, new programs, and new projects that were undertaken at work during her absence. This provided the client a sense of what happened while she had been away to help her feel less “lost” once back at work. The client also created a program proposal and budget that helped re-familiarize her with Excel and its functions. Through these work tasks, the client rebuilt her cognitive abilities (e.g., concentration, attention to detail) and regained self-confidence. Work stamina also increased through graded work tasks and progressive work hours.

Another component of the client's CWH program was coping skill development utilizing professional development videos, role plays and coaching by the occupational therapist. Effective communication strategies, such as assertiveness skills, were targeted for the purpose of interpersonal relationships and enhanced social skills with a view toward helping the client better cope at work thus potentially serving in a preventative role in future sick leave (36). The occupational therapist also equipped the client with strategies to help with her heightened anxiety such as positive self-talk, cognitive reframing, and pacing. Discussions with the therapist rounded out the client's program so that it was a blend of work tasks and skill development.

Consistent with occupational therapy practice and its promotion of health and well-being through occupation, the client's engagement in meaningful activity (outside of work) was monitored through self-reports and discussions with the therapist. This augmented the focus on work functioning by including a more holistic and integrated approach to treatment and RTW preparation.

The client reported generalized neck pain which was associated with time at the computer. The occupational therapist paid attention to the client's workstation ergonomics and ensured that her setup included ideal equipment heights for proper positioning. In addition, the therapist provided education on pacing strategies for pain/fatigue management and education on ergonomic principles to ensure that body biomechanics were addressed with a view toward minimizing pain/discomfort and potential impact on functioning.

**Figure 3:**
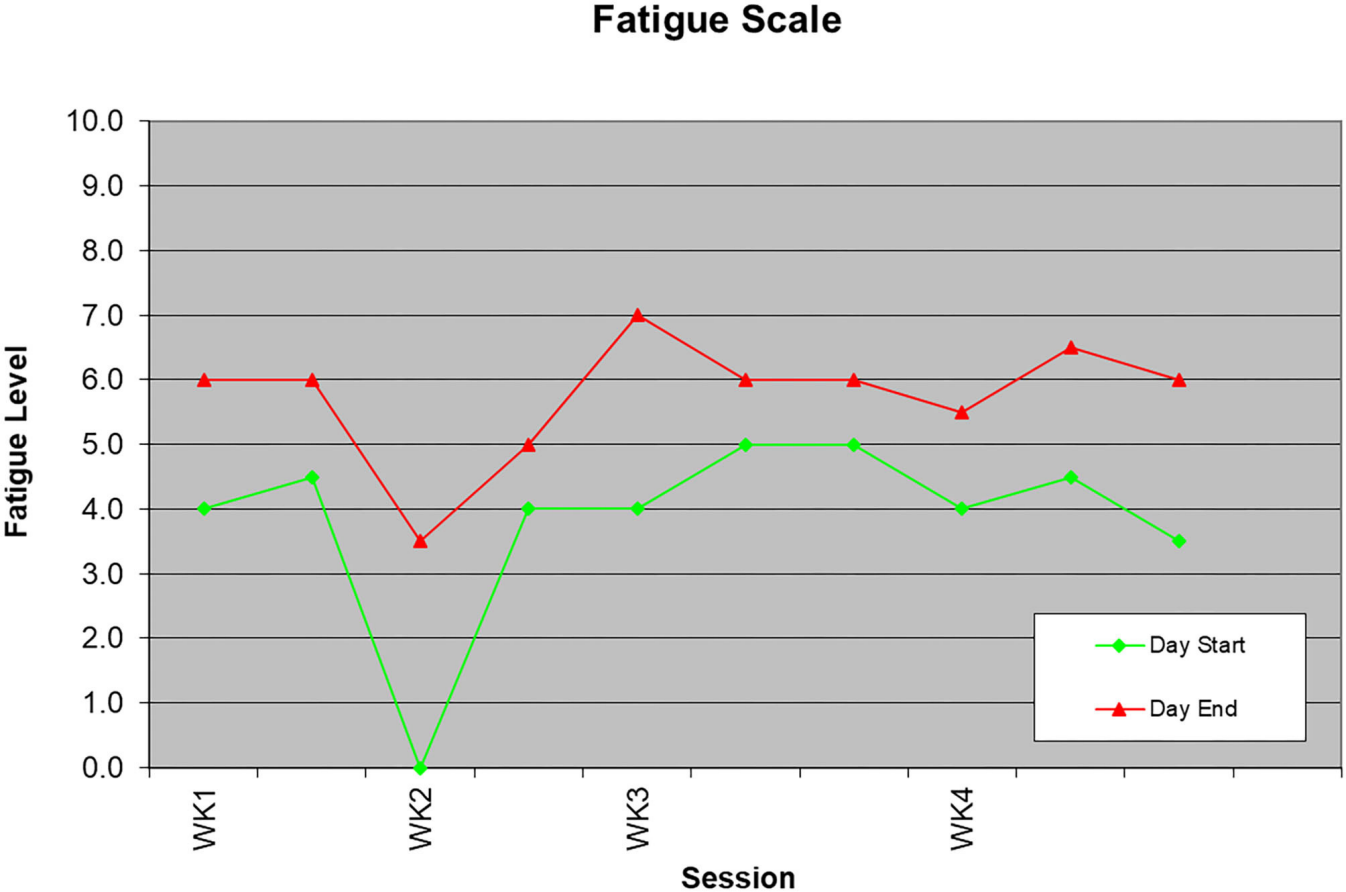
In-session fatigue levels.

In-session metrics included a self-report 10-point fatigue scale to quantify the client's fatigue. Overall fatigue levels were moderate and although there was an increase of one to two levels from start to end of a session (e.g., 4.0–6.0; 5.0–6.0), levels remained in the moderate range (see Figure 3). This pattern remained stable across the 4 weeks even with progression in hours and task complexity. Such a profile suggests that the client was tolerating CWH hours/tasks. Indeed, her work stamina increased from 2-h to 4-h sessions and her weekly work hours increased from 4-h to 12-h with consistent work productivity.

### Intervention Outcome

Assessment of work ability was derived from clinical observations, session tools/metrics, and the City of Toronto's Behavioral/Cognitive Functional Abilities Evaluation (37). The latter includes ratings of 16 behavioral and cognitive abilities (e.g., attention to detail, multitasking) according to clearly-defined criteria. Ratings of five executive skills were added to evaluate goal-directed behavior, problem solving, and emotional regulation.

For example, “ability to self-supervise” is evaluated along four criteria of function: (1) Cannot self-supervise, requires constant work supervision; (2) Requires frequent supervision; (3) Can tolerate infrequent supervision; and (4) Able to carry out work tasks in a self-supervised manner. The client in this report was given a rating of three with the following comment: “The client was able to work independently with minimal supervision once tasks were determined. Within each task, she was able to self-supervise and to progress from beginning to end without ongoing supervision.”

Findings from the client's CWH program revealed no limitations in cognitive abilities that would preclude a return to competitive employment; however, several issues needed to be addressed through accommodations in order to maximize RTW success:

- Reduced work pace pointed to the need for limited multitasking and (tight) deadlines.
- Potential for fatigue required the incorporation of pacing strategies at work including regular breaks, a walk outdoors and healthy eating (e.g., snacks, lunch).
- Reported neck pain/discomfort needed to be addressed through a worksite ergonomic assessment to identify/rectify ergonomic risks (e.g., equipment heights).
- Potential for heightened anxiety (especially with anticipated negative situations and/or confrontation) required the client to use the assertiveness and stress management strategies learned at CWH.

Notwithstanding these issues, the client demonstrated work ability as evidenced in her adoption of a work routine and display of work skills. Through engagement in work simulations, she demonstrated task engagement, sustained concentration, the ability to work in a sequential manner to achieve task goals, and the mental flexibility to respond to task requirements. She was able to work effectively on both structured and non-structured tasks. She demonstrated the ability to learn and to apply learnings to concrete work tasks. Such markers of work performance are consistent with competitive employment and were suggestive of RTW potential.

In order to build on the gains the client made during CWH, the occupational therapist recommended the client follow a gradual RTW (GRTW) schedule upon returning to work with its inherent progressive increase in hours and progressive increase in job duties. The therapist recommended that multitasking and deadlines be avoided during the GRTW and that they be phased in gradually thereafter. Close monitoring was also recommended to ensure the client was coping with the work transition and was able to increase work hours and job demands. Access to job accommodations upon RTW is more typical among employees with high socioeconomic status (SES) and educational level (38) thus positioning this client in a likely advantageous position for RTW success.

Intervention outcome is also noted through the client's pre- and post- questionnaire scores which indicated that her perceived work ability increased, fatigue decreased, and depression symptoms decreased (see Table 1). These quantitative findings aligned with the clinical observations of the client's occupational functioning during CWH.

**Table 1:** Pre-post responses: WAI, MAF, BDI-II.

	**WAI^a^**	**MAF^b^**	**BDI-II^c^**
Program start (pre)	3	31	32
Program end (post)	7	24	20
Outcome at program end	Increased perceived work ability	Decreased fatigue	Decreased depression symptom severity

a *WAI scores range from 0 (completely unable to work) to 10 (work ability at its best)*.

b *MAF scores range from 1 (no fatigue) to 50 (severe fatigue)*.

c *BDI-II scores range from 0 (no depression symptoms) to 63 (severe depression severity). Ranges of depression severity: 0–10 (normal); 11–16 (mild); 17–20 (borderline clinical depression); 21–30 (moderate); 31–40 (severe); over 40 (extreme depression)*.

The increased perceived work ability from 3 to 7 (on a 10-point scale) reflected the client's recognition of the gains she made in cognitive abilities through experiential task mastery which contributed to her sense of efficacy and work ability. Reduced fatigue levels (from 31 to 24) reflected a decrease in the client's fatigue in her personal life (outside of CWH) consistent with her report of increased activity re-engagement. This outcome is noteworthy given that low activity is often a symptom of a depression and has been found to be predictive of repeated sick leave in a population of depressed patients (39). Depression severity decreased from a score of 32 (“severe” depression) to 20 (“borderline” clinical depression). This aligned with the client's overall positive outlook to RTW by the end of her CWH program.

### Follow Up

Three months after program completion, the client participated in a semi-structured phone interview with the occupational therapist. This follow-up interview provided the client the opportunity to reflect on her experience of the intervention and to consider that experience in relation to her own RTW trajectory. The interview protocol was two-fold. First, it consisted of directed questions regarding the client's employment status to determine if she had returned to work or if she had remained on disability. Indeed this is considered to be the primary measure of RTW success (21). If working, the client was asked to rate her sense of well-being at work along three pre-defined categories: (1) working/many issues (struggling); (2) working/some issues (coping); and (3) working/few or no issues (doing well) (22). The second aspect of the protocol consisted of open-ended directed questions regarding the client's perceptions of and experiences with the CWH intervention given the time lag since participation in the intervention. This included inquiry into how the CWH intervention helped her prepare for RTW, what elements of the CWH intervention she found helpful, and if there were any aspects of the intervention she did not find useful or would have liked done differently.

The client reported that she was at work “doing well.” She noted that when she returned to work (after CWH), she adhered to an 8 week GRTW schedule which started at 8 h/week with a progressive increase in hours from week to week. At follow up, she reported to be working full time hours assuming a full workload with good stamina and concentration. She shared that the CWH intervention had been helpful and indeed prepared her to return to her pre-disability job. The client noted that the CWH experience had given her increased self-confidence and that the coping skills she had learned were indeed helpful once she was back at work. She also reported that she was seeing her psychiatrist every 3 months and no longer saw her psychologist.

## Discussion

This case report illustrates the application of CWH to facilitate RTW following depression through a real life example of the workings of the intervention. The specific CWH intervention utilized was pioneered and developed at ERGO-Wise, an occupational therapy private practice in Ottawa, Canada. Scientific study of this CWH intervention supported its role in RTW following depression (21, 22).

The first-hand account of the functional challenges facing a client returning to work following depression provides insight into the myriad occupational challenges facing this population and the application of CWH to target specific issues. This case demonstrates how CWH is aptly positioned to respond to the call for treatment interventions that augment reduction in depressive symptom severity and specifically target occupational function for people with depression (6, 10, 12, 40). This is especially relevant for knowledge workers whose job demands are more intellectual and psychosocial in nature (41). Moreover, this case report illustrates how to apply the multiple CWH elements (42) in a very direct and concrete manner thus providing a roadmap to guide practitioners toward enhanced RTW outcomes. Elements were discussed individually as well as how they combined to address the full array of RTW challenges.

The report also shows how the customization of CWH is essential to maximize RTW preparation. Indeed the client explicitly remarked on the CWH plan having been specifically designed for her needs and RTW goals. Through relevance of work simulations, individualization of coping skill development, and personalized computer skill retooling, all against the backdrop of a simulated work environment, the client recognizes work skills and anticipates their transfer to the actual workplace. Increased self-confidence and feelings of self-efficacy typically ensue which facilitate movement from a “patient identity” to a “worker identity,” shown to be an underlying factor of RTW success (43). The client captured this in her perspective along with noting the importance of establishing a daily routine—another component of CWH's RTW preparation and precursor to RTW.

In addition to preparing clients to return to work, the CWH process provides objective data regarding work ability including strengths, limitations, and restrictions that help guide RTW planning and identify any job accommodations required to maximize RTW success. For example, directly observing the impact of the client's fatigue on work pace and the critical role that pacing strategies played in fatigue management while at CWH provided confirmation for their importance once back at work with anticipated increase in work hours and job demands. Task engagement reflected the client's response to work demands. Limited ability to multitask and tolerate deadlines at CWH highlighted the necessity for a gradual reintegration into the workplace with a phasing in of these job requirements to facilitate RTW transition. Pain associated with poor positioning highlighted the need for sound ergonomics through workstation adjustments. Finally, the client's need to apply assertiveness and stress management strategies was evident in her reporting of heightened anxiety at different times during CWH in response to task, interpersonal situations, and anticipated work scenarios. The “real time” responses to the work demands of the CWH intervention provided invaluable information regarding the client's work functioning which equipped her with RTW strategies and drove the RTW planning process.

As is often the case with the case report genre, limitations of this report include lacking generalizability, inability to show cause and effect, and the danger of over interpretation of a single case. That said, the case report is a strong vehicle to present the novel (CWH) approach, offer a valuable in-depth lens into CWH, and complement the empirical research. With respect to the intervention itself, incorporating targeted standardized cognitive tests may be a useful complement to the current measures and tools already in place. Future studies on other CWH interventions would enhance our understanding of this intervention and further establish its role in RTW. Longer follow up is needed to determine the more long-term effects of CWH.

It is noted that the client presented in this case report was a knowledge worker with a high educational level and at least moderate SES. These two factors have been found to likely increase access to treatment interventions which can facilitate RTW success (38). Furthermore, the client presented was returning to her permanent pre-disability job which offered her some level of security while being on sick leave and while preparing to return to work. Indeed, temporary employment has been associated with slower RTW after a disability leave (44). These factors need to be considered as possible positive prognostic indicators for success.

This case report builds on prior research by providing a window into the application of CWH within a real world context. In particular, it shows how the intervention elements are used to address a specific client's issues leading to a successful RTW outcome. The underlying theory of change and intervention elements can be applicable to other RTW interventions with implications for their treatment goals. The report adds to the body of literature on RTW, rehabilitation, and the occupational therapy field.

## Patient Perspective

I received a telephone call from the insurer that it was time for me to return to work. Immediately I was flooded with anxieties regarding how I was going to cope—the fears just kept escalating. Then I received a telephone call telling me about cognitive work hardening, what it could do for me prior to returning to work, and if I was interested—I was!

The occupational therapist met with me personally discussing my absence from work, how I felt about returning to work and then worked out a plan specifically for me to help with my return to work. I established a daily routine, getting up as if I was going to work and returning home. The therapist stressed the necessity of taking breaks, time out, eating and the ability to interact with other people if I wanted to. I was provided with an office work environment where I could work on mind challenging tasks exercising my memory, problem solving ability, and reading comprehension. I had the use of a computer to re-tool my skills. Through these experiences my mind was excited and triggered. I gained logical thinking and confidence. Completing my job description reminded me of my job responsibilities and functions, again providing me with confidence and a sense of “I can do this.”

I was given the opportunity to talk about how I was doing and to review my work tasks. I shared my fears of returning to work and received guidance on ways to think through my thoughts. I gained confidence and a better way of thinking that included not to stress about the past, not to stress about the future—it isn't here yet. I emerged with a sense of living day to day and believing that I can do what I need to do! Thank you. I am so grateful.

## Data Availability Statement

The original contributions presented in the study are included in the article/Supplementary Material, further inquiries can be directed to the corresponding author/s.

## Informed Consent

The client presented in this report provided written informed consent for the publication of this case report.

## Author Contributions

AW was the treating occupational therapist, observed the client, collected and analyzed the data and wrote the paper.

## Conflict of Interest

The author declares that the research was conducted in the absence of any commercial or financial relationships that could be construed as a potential conflict of interest.

## Footnotes

^1^Results of the BDI-II provided context to occupational performance and were not intended for diagnostic purposes.
